# Effect of ZrC on the Microstructure and Properties of CrMnFeCoNi High-Entropy Alloy Coatings Prepared by a Plasma Transferred Arc Process

**DOI:** 10.3390/ma16237401

**Published:** 2023-11-28

**Authors:** Long Huang, Bingyuan Li, Bopin Xu, Yicheng Zhou, Mengzhao Li, Chenglin Li, Bing Yang, Chunxu Pan, Guodong Zhang

**Affiliations:** 1 School of Power and Mechanical Engineering, Wuhan University, Wuhan 430072, China; 2 Hubei Port Yakou Navigation Junction Limited Company, Xiangyang 441100, China; 3 School of Physical and Technology, Wuhan University, Wuhan 430072, China

**Keywords:** high-entropy alloy, ZrC, plasma transferred arc, microstructure, properties

## Abstract

The low strength caused by the single FCC structure of the CrMnFeCoNi high entropy alloy (HEA) limits its application in the field of coating. Here, we prepared high-entropy alloy coatings of CrMnFeCoNi with different ZrC contents on Q235 steel by a plasma transferred arc process. The effects of ZrC on the microstructure and properties of the CrMnFeCoNi HEA coating were investigated by optical microscopy, scanning electron microscope, and X-ray diffraction and by employing a potensiostat/galvanostat. The results showed that ZrC mainly existed in the coatings as a second phase, having little influence on the main crystal structure and micromorphology of the CrMnFeCoNi HEA coating. The hardness of the CrMnFeCoNi HEA coating increased with the ZrC content. ZrC can effectively improve the corrosion resistance of the CrMnFeCoNi HEA coating. In a 1 mol/L NaCl solution with 4 wt% ZrC, the annual corrosion rate was only 5.997% of that of the HEA coating. Nevertheless, the improvement in the wear resistance of CrMnFeCoNi high-entropy alloy coatings was not apparent with the addition of ZrC. Consequently, the addition of ZrC to the FeCoCrNiMn high-entropy alloy coating holds promise for applications in corrosion resistance, particularly in oceanic environments.

## 1. Introduction

With the progress of society and the development of technology, the requirements for materials are increasing, and traditional alloy materials can no longer meet market needs. In 2004, the scholar Yeh introduced the concept of a high-entropy alloy (HEA) in Advanced Engineering Materials. HEAs were initially defined as alloys composed of five or more principal elements in equimolar or near-isomolar ratios [1]. In the past 20 years, with the in-depth study of HEAs, certain alloys formed with quaternary nonisomolar ratios have also been defined as HEAs [2]. The emergence of HEAs has changed the understanding of alloys based on the Gibbs phase rule. Experiments by Canto and Yeh showed that alloys with equimolar ratios of multiple components tend to form solid solutions with simple structures [3,4]. The microstructures of HEAs were shown to consist of randomly distributed and disordered atoms at the lattice positions. It was also shown that HEAs have a high-entropy thermodynamic effect, a structural lattice distortion effect, a sluggish diffusion dynamics effect, and a cocktail performance effect [5]. As a result, HEAs have the characteristics of high strength, high hardness, wear resistance, corrosion resistance, and high-temperature resistance, which traditional alloys cannot match. Refractory high-entropy alloys, which include elements such as Mo, Nb, Ta, V, and W, are considered to be promising materials for high-temperature applications. These alloys are suitable for use in atomic energy, aerospace, military, and advanced nuclear reactors [6]. The high-entropy alloy containing Cr, Ni, and Mo has an excellent corrosion resistance and can be used in the marine field [7]. High-entropy alloys can be utilized in biomedical applications due to their exceptional biocompatibility achieved through composition design [8,9].

Although HEAs have a great application potential in many fields due to their excellent physical and chemical properties, the high cost of HEA preparation limits their widespread use. The idea of using HEAs as coatings provides a direction for HEA applications. For example, corrosion-resistant HEA coatings can be applied in the ocean, high-pressure-resistant HEA coatings can be applied in oil and gas, high-temperature-resistant HEA coatings can be applied in aerospace and nuclear energy fields, etc., [10,11]. At present, the preparations used for HEA coatings include mechanical alloying [12,13], thermal spraying [14,15], cold spraying [16,17,18], laser cladding [19,20,21,22,23,24,25,26,27], magnetron sputtering [28,29,30], and plasma transferred arc (PTA) [31,32,33,34,35,36]. Among them, PTA has the characteristics of simple equipment, high production efficiency, concentrated energy, less environmental effects, and metallurgical bonding with the substrate, which are beneficial for forming high-quality coatings. Therefore, the PTA has been widely used in coating research.

CrMnFeCoNi, as a typical HEA with a simple face-centered cubic (FCC) structure, is one of the main research objects for studying HEAs. Because of its simple FCC structure, the CrMnFeCoNi HEA has the characteristics of high toughness and low strength. Therefore, it is necessary to strengthen the CrMnFeCoNi HEA when used as a coating. ZrC, as a high-temperature ceramic material with high strength, good corrosion resistance, and chemical stability, has been widely used in material strengthening. To investigate the influence of ZrC on the corrosion resistance and wear resistance of Ni-P coatings, He et al. [37] added ZrC to a Ni-P nanocomposite coating on N80 steel by chemical deposition. The results showed that ZrC effectively reduced the friction coefficient, wear rate, and coating defects to improve the corrosion and wear resistance of the coating. Kang et al. [38] added ZrC to Ti(C, N)-based cermets by a sintering process to study the effect of ZrC on the microstructure and properties. They found that adding ZrC increased the wear-resistant carbide and inhibited the precipitation of the brittle phase, thus improving the mechanical properties and oxidation resistance of the cermets. Ding et al. [39] studied the effect of ZrC on the microstructure and mechanical properties of FeCrAl alloys and prepared FeCrAl alloys with different ZrC contents by spark plasma sintering. They found that an appropriate amount of ZrC (1 wt%) effectively refined the FeCrAl alloy grains, thereby improving the strength and hardness of the FeCrAl alloy. However, there are few studies about the effect of ZrC on HEA coatings. In this paper, CrMnFeCoNi HEA coatings with different ZrC contents were prepared on Q235 steel by a PTA process, and the effects of ZrC on the morphology, composition, hardness, friction, and corrosion properties of the coatings were investigated.

## 2. Materials and Methods

### 2.1. Material Preparation

**Figure 1:**
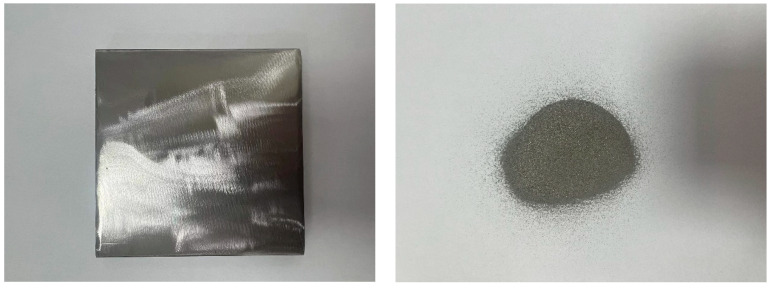
Pictures of the substrate and powder.

Figure 1 shows a picture of the substrate and powder. The substrate material of this experiment was 100 × 100 × 10 mm Q235A steel. The chemical composition of the Q235A steel is shown in Table 1. This steel is a commonly used engineering structural steel with an excellent welding performance. The HEA powder used for this experiment was CrMnFeCoNi, prepared by Beijing Yanbang New Materials Co., Ltd. (Beijing, China). Its particle size was 50–150 μm. The chemical composition of the CrMnFeCoNi HEA is shown in Table 2. Due to the low fluidity of the irregular ZrC powder, excessive ZrC would easily block the feeding pipe, so the maximum addition of ZrC was set as 4 wt%. The HEA powder and the ZrC powder were uniformly mixed by ball milling to form HEA and x wt% ZrC coatings (x = 1, 2, 3, 4; denoted as 01ZrC, 02ZrC, 03ZrC, 04ZrC).

### 2.2. Experimental Method

**Figure 2:**
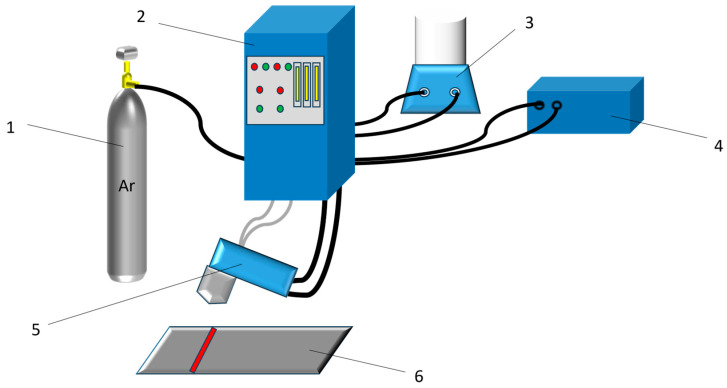
The sketch of the PTA equipment. 1: Powder feed gas and shielding gas; 2: Control system; 3: Powder feeding equipment; 4: Cooling system; 5: Welding torch; 6: Substrate.

**Figure 3:**
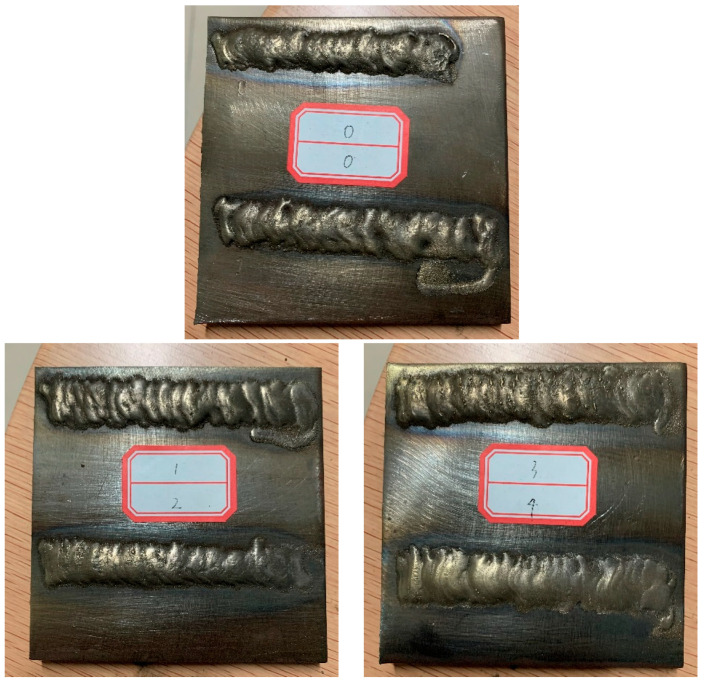
Picture of HEA and XZrC coatings: (0) HEA; (1) 01ZrC; (2) 02ZrC; (3) 03ZrC; (4) 04ZrC.

A PTA-BX-400A powder plasma surfacing machine (manufactured by Shanghai Benxi Electromechanical Technology Co., Ltd., Shanghai, China) was used to prepare XZrC (X = 01, 02, 03, 04) coatings onto impurity-free Q235 steel. The sketch of the PTA equipment is shown in Figure 2. High-purity argon was used as the powder feed gas and the shielding gas. The coating was manually prepared to a thickness of approximately 2.5 mm. The process parameters are shown in Table 3. Pictures of HEA and XZrC coatings are shown in Figure 3. 

The sample was cut into 10 × 10 × 10 mm cubes and Φ 8 × 10 mm cylinders with a wire electrical discharge machine. The surfaces of the cubes to be measured were ground, polished, and corroded. An LAB-1 optical microscope (OM, manufactured by Olympus Corporation, Tokyo, Japan) was used to observe the metallographic structure. A MIRA 3 LMH field emission scanning electron microscope (SEM, manufactured by TESCAN Brno, s.r.o., Brno, Czech Republic) and an Aztec Energy X Max 20 energy dispersive spectrometer (EDS, manufactured by Oxford Nanoimaging, Oxford, Britain) were used to observe the microstructure and elemental distribution of the coatings. 

A Bruker D8 X-ray diffraction (XRD, manufactured by Bruker Corporation, Billerica, MA, USA) instrument was used to analyze the phase composition and crystal lattice structure of the coatings. The characteristic wavelength was 1.54060 Å, the voltage was 60 kV, the scanning angle was 10° to 90°, and the scanning speed was 2°/min.

An HXS-1000 A microhardness tester (manufactured by Shanghai milite Precise Instrument Co., Ltd., Shanghai, China) was used to measure the hardness of the coatings. The average value of 5 points was taken every 100 μm from the bottom of the coating. The load was 300 g and it was applied for a duration of 10 s.

**Figure 4:**
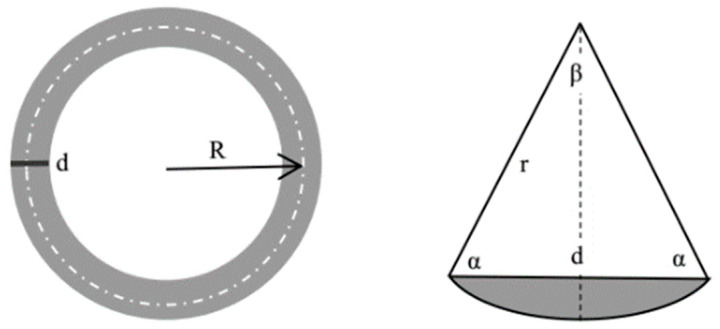
Principle of material volume loss calculation.

An MS-T3001 friction and wear tester (manufactured by Lanzhou Huahui Instrument Technology Co., Ltd., Lanzhou, China) was used for the wear test of coatings. The wear mode was Pin-on-Disk (ASTM G99-04 [40]). The friction pair was SUS304 stainless steel, which had a hardness of approximately 320 HV and a diameter of 3 mm. Table 4 shows the parameters of the wear experiment. The samples were ground to a roughness of 3.5 μm. The experiments were repeated with three samples. SEM was used to observe the wear morphology of the samples, and EDS was used for quantitative analysis. The volume loss was calculated based on the width of the wear track. The principle of material volume loss calculation is shown in Figure 4. The specific calculation equation is as follows [41]:

(1) $$V_{loss}=C\times S$$

(2) C=2πR

(3) $$S=\left(\frac{\pi r^{2}\beta }{2\pi }-\frac{1}{2}d\sqrt{r^{2}-{\left(\frac{d}{2}\right)}^{2}}\right)$$

(4) $$\beta =\pi -2\arccos\left(\frac{d}{2r}\right)$$

(5) $$V_{loss}=2\pi R\left(\frac{\pi r^{2}\beta }{2\pi }-\frac{1}{2}d\sqrt{r^{2}-{\left(\frac{d}{2}\right)}^{2}}\right)$$

where $V_{loss}$ is the material volume loss (mm^3^); *C* is the wear circumference (mm); *S* is the wear cross-sectional area (mm^2^); β is the arc angle of the friction; *d* is the width of wear track (mm); *r* is the radius of the counterpart (mm); *R* is the radius of the wear track (mm).

To evaluate the corrosion resistance of the coatings, electrochemical impedance spectroscopy (EIS) and polarization curves were obtained using a CS310H potentiostat/galvanostat. A 1 mol/L NaCl solution was used as the corrosion medium. The reference electrode used was a saturated calomel electrode (SCE). The counter electrode was platinum. In this study, the samples were Φ 8 × 10 mm cylinders, and the round exposed surface was polished. The electrochemical experimental parameters are shown in Table 5. The electrochemical experiment was performed three times with nominally identical samples.

## 3. Results and Discussion

### 3.1. Phase Composition of the Coating

**Figure 5:**
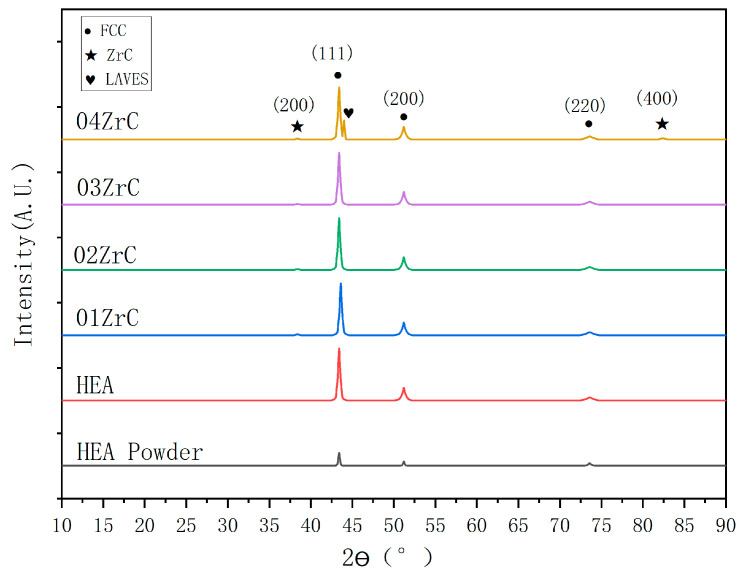
XRD pattern of the CrMnFeCoNi and XZrC high-entropy alloy coatings.

Figure 5 shows the XRD patterns of the powder of HEA and XZrC (X = 00, 01, 02, 03, 04) coatings. It can be observed that the peak strength of the HEA powder is significantly lower than that of the coating, which could be related to the condition of the material. Their main diffraction peaks are (111), (200), and (220), indicating that the HEA powder has been mechanically alloyed. Furthermore, the main crystal structures of the HEA powder and all five HEA coatings are face-centered cubic (FCC). Therefore, adding ZrC does not change the CrMnFeCoNi HEA coating structure. Among these diffraction peaks, the peak of the (111) crystal plane has the highest diffraction intensity, which means that the preferred growth direction during solidification is in the direction of the (111) crystal plane. When the ZrC content increased to 4 wt.%, a splitting peak appeared near (111). This may be due to the fact that a sufficient amount of ZrC increases the content of dissolved Zr, which forms a Laves phase with matrix elements [42]. Table 6 shows the composition of the phases in the HEA powder and the five coatings calculated by Jade6. It can be observed that the phase content of ZrC gradually increases with the addition of ZrC. By 04ZrC, the Laves phase was present.

According to the Scherrer formula, the crystallite sizes of the FCC of the HEA powder and the 00–04ZrC coating are 39.5 nm, 19.3 nm, 18.6 nm, 18.3 nm, 18.2 nm, and 18.0 nm, respectively. It shows that the rapid cooling of PTA reduces the crystallite size of HEA compared to HEA powder. In addition, the crystallite size decreases with the increase in ZrC content. This is due to the fact that the addition of the second phase hinders grain growth, resulting in a gradual reduction in the crystallite size of FCC. However, the addition of ZrC has little effect on the crystallite size of FCC. The crystallite sizes of ZrC in 01–04ZrC are 11.1 nm, 12.7 nm, 11.5 nm, and 12.2 nm, respectively. These sizes show little change, likely due to the consistent influence of the same process parameters on ZrC. The crystallite size of the Laves phase precipitated in 04ZrC is 11.8 nm.

The lattice constants of the HEA powder and the 00–04ZrC coatings calculated by Jade6 are 0.35918 nm, 0.3596 nm, 0.3578 nm, 0.3604 nm, 0.3612 nm, and 0.3606 nm, respectively, which suggests that the addition of ZrC has little effect on the lattice constants of HEA coatings. The explanation for this is that due to the high melting point of ZrC, most of the ZrC particles do not decompose but exist as a second phase in the HEA coatings. As a result, there is no significant change in the lattice constants of the HEA coatings.

### 3.2. Morphology and Composition Analysis of the Coatings

**Figure 6:**
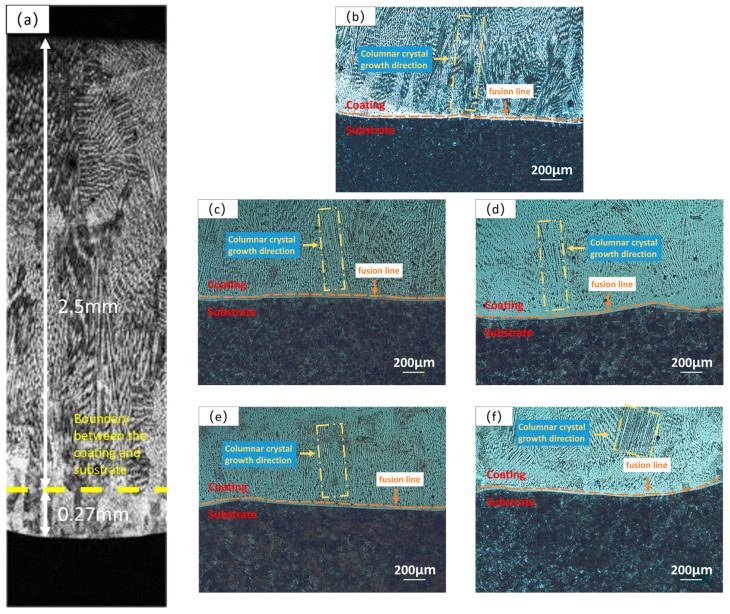
Microstructure of the HEA and XZrC coatings. (**a**) Cross-section of the entire thickness of the coating; (**b**) HEA; (**c**) 01ZrC; (**d**) 02ZrC; (**e**) 03ZrC; (**f**) 04ZrC.

Figure 6 shows the metallography of the HEA and XZrC (X = 0.1, 0.2, 0.3, 0.4) coatings near the fusion line observed by optical microscopy. The thickness of the coatings is 2.5 mm, and the melting depth is 0.3 mm. The calculated dilution rate is 10.71%, indicating a strong metallurgical bond between the coating and the substrate (Figure 6a). Furthermore, the five coatings were composed of dendrites and equiaxed crystals perpendicular to the matrix. This is due to the fact that during the solidification process of the coatings, the heat loss was slower in the area near the substrate than that near the air, which resulted in a temperature gradient that made the columnar crystals grow along the direction perpendicular to the substrate. Therefore, the addition of ZrC had little effect on the microstructure of the coatings. 

**Figure 7:**
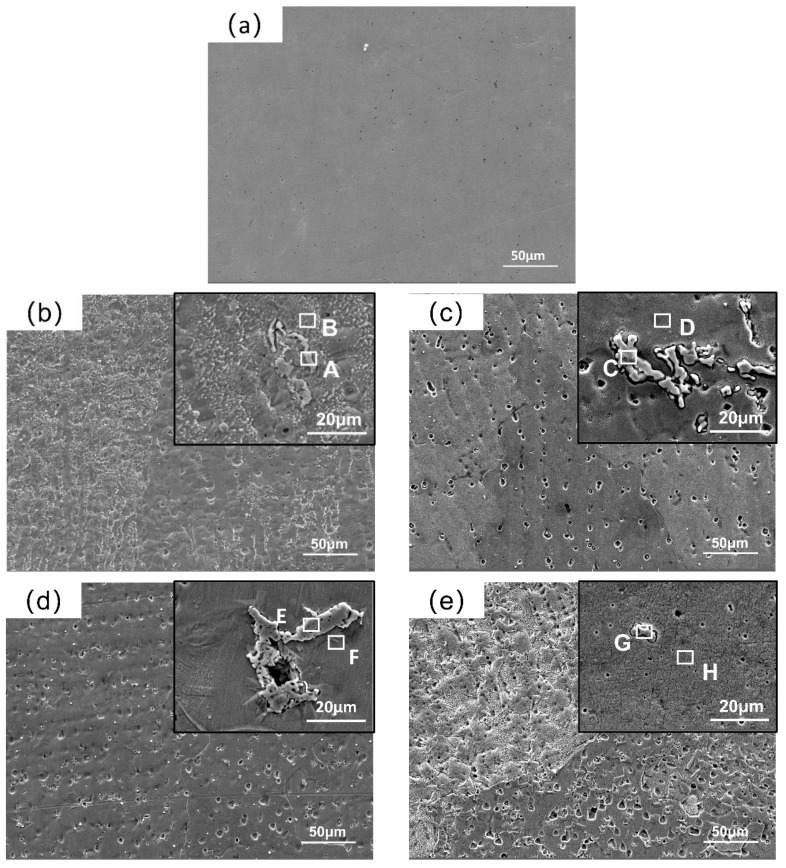
SEM images of the XZrC coatings. (**a**) 00ZrC; (**b**) 01ZrC; (**c**) 02ZrC; (**d**) 03ZrC; (**e**) 04ZrC.

Figure 7 shows the SEM images of the XZrC HEA coatings. The diameter and density of the pits increased with increasing ZrC content. It is speculated that the unmelted ZrC particles on the surface of the coatings may cause flaking during grinding and polishing [43]. In addition, with increasing ZrC content, the number of spalling ZrC particles increases. Scratches that may be produced by the exfoliation of ZrC particles are also observed in the high-magnification SEM image of the 04ZrC coating. In addition, granular precipitates are observed in the high-magnification SEM images of the 01–04ZrC coatings, indicating that ZrC may exist in the CrMnFeCoNi HEA as a second phase [44]. In the high-magnification SEM image, it can also be seen that the size of the precipitates is quite different, which also indicates that the low fluidity of ZrC causes its uneven deposition in the coating. In addition, perhaps due to the too-small particle size of the generated Laves, the Laves was not seen in the SEM image of 04ZrC.

To clarify the specific composition of the granular precipitates, the precipitates in the 01–04ZrC coatings and the area surrounding the precipitates were analyzed by EDS. The results are shown in Table 7. The four points of A, C, E, and G indicate areas of the precipitate that are mainly Zr, as well as a few elements of the CrMnFeCoNi HEA, which verifies that the main component of the granular precipitate is unmelted ZrC. The main elements of the points B, D, F, and H of the area near the precipitates are CrMnFeCoNi HEA principal elements, and the contents of the five principal elements are close to equal. 

### 3.3. Microhardness

**Figure 8:**
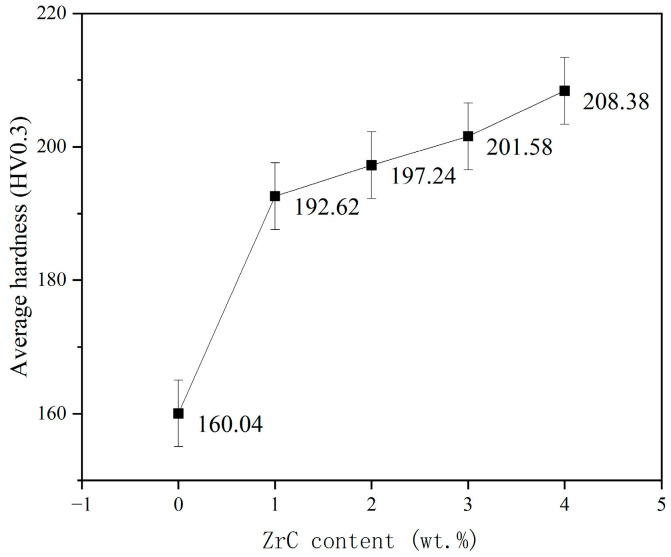
Average hardness of the HEA and XZrC coatings.

Figure 8 shows the average hardness of the HEA and XZrC (X = 01, 02, 03, 04) coatings. The hardness of the CrMnFeCoNi HEA coating without ZrC is very low, with an average hardness of only 160.04 HV. This is mainly because the principal elements of CrMnFeCoNi HEAs are adjacent in the periodic table, which is indicative of their similar atomic sizes. Therefore, there is no significant lattice distortion in the crystal structure, leading to the low hardness of the CrMnFeCoNi HEA coating. With increasing ZrC content, the hardness of the HEA coating gradually increases. The hardness increases significantly at the beginning of ZrC addition. The figure shows that the hardness of 01ZrC increases by approximately 20% compared to that of 00ZrC. Subsequently, the hardness increases more slowly with increasing ZrC content. The main reason for this is that when ZrC is newly added to the CrMnFeCoNi HEA coating, ZrC is deposited as a hard phase and introduces a second-phase strengthening mechanism, resulting in a significant increase in hardness [32]. Then, the strengthening mechanism does not change with increasing ZrC content. Only the amount of the second phase and dissolved ZrC content were increased, and the increase was slight, leading to a slower increasing hardness trend.

### 3.4. Friction and Wear Properties

**Figure 9:**
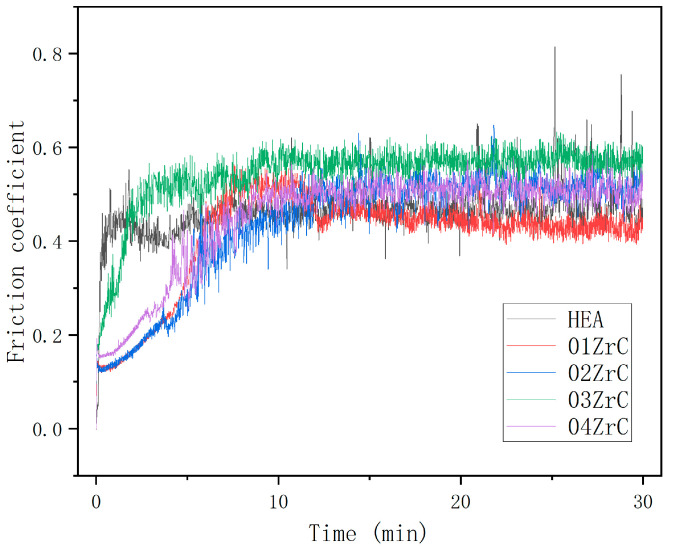
Friction coefficient of the high-entropy alloy coatings with different ZrC contents.

After conducting the rotational friction experiments, the friction coefficient curves of HEA and XZrC are shown in Figure 9. It can be observed that the five coatings experienced two stages of adaptive friction and stable friction during the experiment. This indicates that the friction mechanism of the five coatings is similar. Among them, the HEA friction coefficient curve reaches the stable friction stage quickly and fluctuates significantly. This may be due to the significant difference in hardness between the friction pair and the HEA. Due to the addition of ZrC, the hardness of the HEA coating is significantly increased. As a result, the friction coefficient curve of XZrC grows more slowly and has a smaller fluctuation range. The friction coefficient of 01ZrC in the stable friction stage is the smallest, while that of 03ZrC is the largest. It can be preliminarily judged that 01ZrC exhibits the best wear resistance, while 03ZrC demonstrates the worst wear resistance.

**Figure 10:**
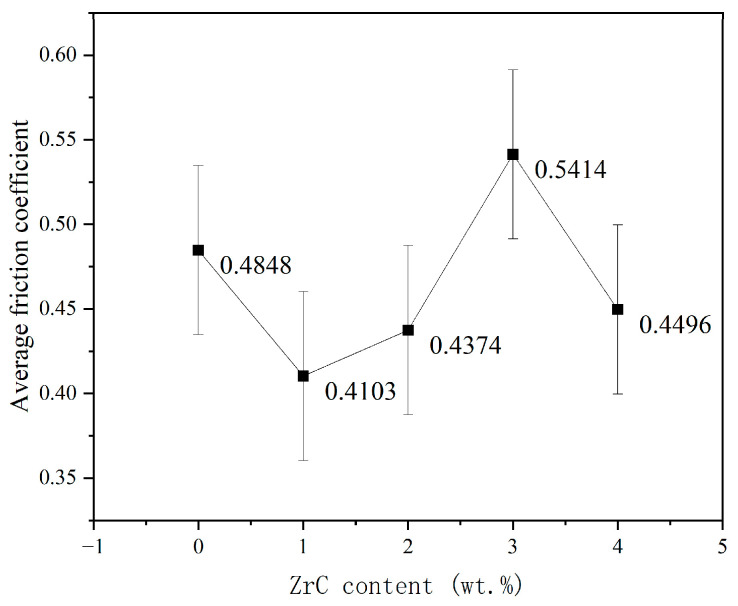
Average friction coefficient of the high-entropy alloy coatings with different ZrC contents.

The curve of the average friction coefficient changing with the content of ZrC was obtained as shown in Figure 10. The average friction coefficient is calculated at the stable friction phase. With increasing ZrC content, the average friction coefficient of the coatings shows the trend of first decreasing, then increasing, and then decreasing again. This indicates that the addition of the appropriate amount of ZrC can reduce the friction coefficient to improve the friction performance of the HEA coating. Among the different coatings, the 01ZrC coating has the smallest friction coefficient, which is reduced by approximately 15.4% compared to that of the HEA without ZrC. When the content of ZrC is 3 wt%, the friction coefficient reaches a maximum and even surpasses that of the pure HEA.

**Figure 11:**
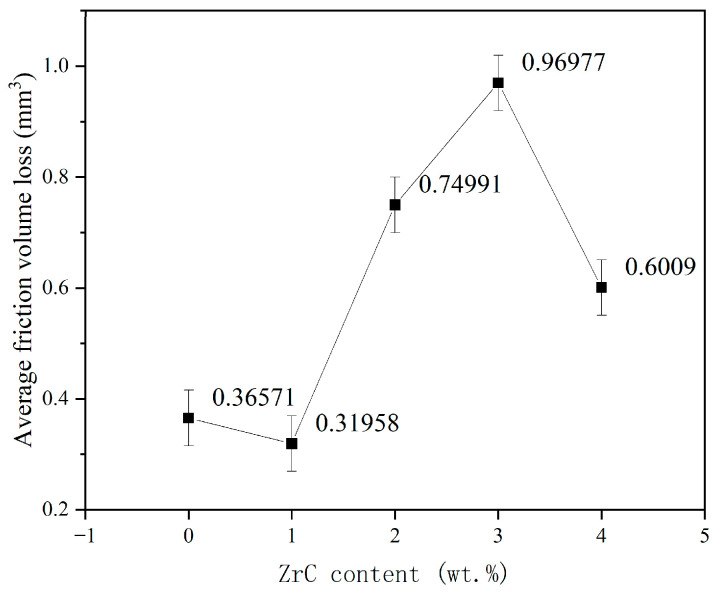
Average friction volume loss of the high-entropy alloy coatings with different ZrC contents.

Figure 11 shows the curve of friction volume loss of HEA and X ZrC, and it can be seen that its variation trend is consistent with the average friction coefficient. The volume loss is the smallest at 01ZrC, and the volume loss is reduced by 12.6% compared with the coating without ZrC. The reduction effect is not obvious. With the increase in ZrC content, the volume loss increases greatly and reaches the maximum at 03ZrC, which is about 2.5 times that of HEA. By 04ZrC, the volume loss is significantly reduced, 38% lower than 03ZrC, but still higher than HEA.

**Figure 12:**
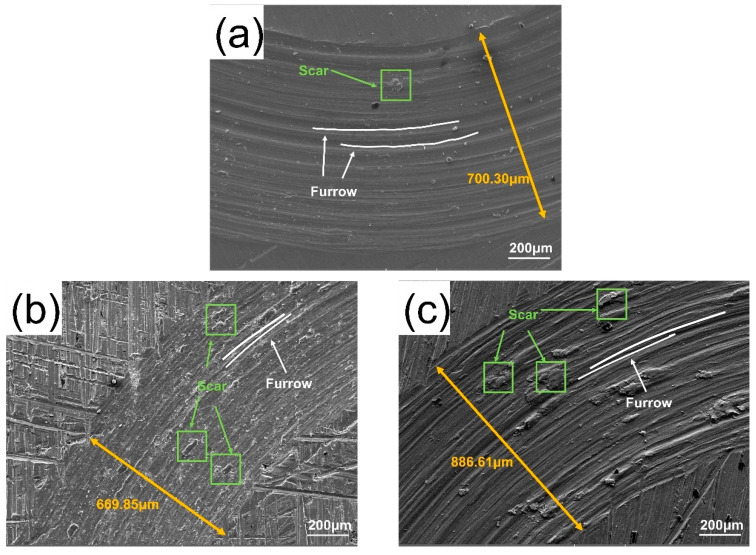

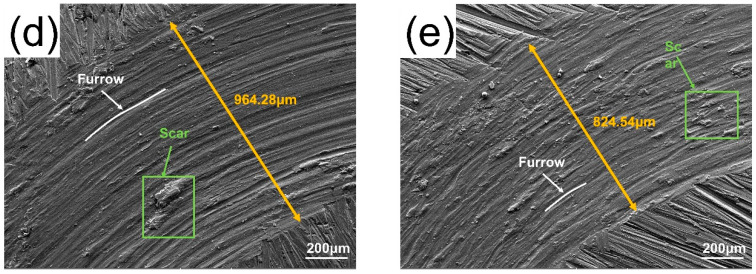
SEM images of the wear marks of the high-entropy alloy coatings with different ZrC contents: (**a**) HEA; (**b**) 01ZrC; (**c**) 02ZrC; (**d**) 03ZrC; (**e**) 04ZrC.

To understand the reasons for this trend, the wear morphologies of the HEA and XZrC (X = 01, 02, 03, 04) coatings were analyzed. Figure 12 shows the SEM images of the HEA and XZrC coating wear marks. The width of the wear mark of 01ZrC was narrower than that of the HEA without ZrC. When the content of ZrC was between 1 wt% and 3 wt%, the width of the wear mark increased gradually with increasing ZrC content. The wear mark of the 04ZrC coating was narrower than that of 03ZrC. It demonstrated that with increasing ZrC content, the wear volume of the coating decreased first, then increased, and then decreased, which was consistent with the trend of the friction coefficient and volume loss. Scars caused by adhesive wear and furrows caused by abrasive wear appeared in all of the five coating wear morphologies. This showed that the wear mode of the coatings was a combination of adhesive wear and abrasive wear. EDS analysis of the scar area revealed that the oxygen content was all approximately 30 wt%, suggesting that tribo-oxidation occurred during the wear process. Usually, tribo-oxidation has a wear-reduction mechanism [45]. Therefore, the more scar content, the better the wear resistance. Compared with HEA, the number of scars in the 01ZrC coating was greater, while the width of the wear mark was narrower, which indicated that the proportion of adhesive wear increased and the friction resistance was enhanced. This was because introducing a second phase dramatically increased the hardness of the coating, thus enhancing the wear resistance. In 01ZrC–03ZrC, with increasing ZrC content, the width of the wear mark and depth of the furrow gradually increased while the number of scars gradually decreased. This indicated that with increased Zr content, the abrasive wear became increasingly severe, and the friction resistance gradually lessened. This was because with increasing Zr content, the number of ZrC particles shed during the wear process gradually increased. These shed ZrC particles were present between the coating and the stainless steel ball, and they increased the proportion of abrasive wear and reduced the friction resistance of the coating. For 04ZrC, the width of the wear mark and the depths of furrows decreased, and the number of scars increased, which indicated enhanced friction resistance. This is due to the strengthening effect of newly generated intermetallic compounds’ Laves, which increase the wear resistance of the coating [46].

### 3.5. Corrosion Resistance of the Coating

**Figure 13:**
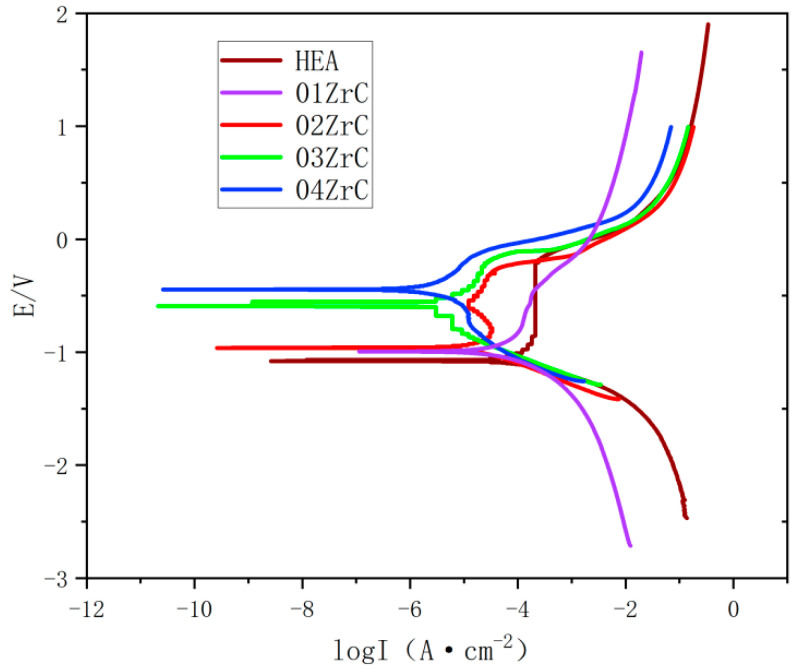
Polarization curves of the high-entropy alloy coatings with different ZrC contents.

The corrosion performance of the HEA and XZrC (X = 01, 02, 03, 04) coatings was investigated by electrochemical experiments with a corrosion medium of 1 mol/L NaCl. Figure 13 shows the polarization curves of the experiments. The shapes of the polarization curves of the five coatings are roughly the same. They indicate that the corrosion forms and mechanisms of these coatings in 1 mol/L NaCl solution are the same. Moreover, the self-corrosion potentials of the 03ZrC and 04ZrC coatings are significantly higher than that of the HEA. It can be preliminarily concluded that the high content of ZrC can significantly enhance the corrosion resistance of the CrMnFeCoNi HEA coating.

In order to determine the specific effect of ZrC on the corrosion resistance of the CrMnFeCoNi HEA coating, the strong polarization region of the polarization curve was analyzed using the polarization resistance (Rp) fitting method with the data analysis module of the potensiostat/galvanostat, and the results are shown in Table 8. With the increase in ZrC content, the polarization resistance (Rp) and self-corrosion potential (E_corr_) gradually increase, while the self-corrosion current density (I_corr_) gradually decreases. This indicates that it is increasingly difficult for the coatings to lose electrons as the ZrC content increases. As a result, the corrosion resistance is gradually enhanced. In addition, the decreasing trend of the self-corrosion potential and self-corrosion current density accelerates significantly when the ZrC content exceeds 2 wt%. Furthermore, the annual corrosion rate gradually decreases with increasing ZrC content. The annual corrosion rate of the 04ZrC coating in a 1 mol/L NaCl solution is only 5.997% of that of the HEA. This indicates that the corrosion resistance is significantly improved. The reason for this is that the unmelted ZrC particles are deposited in the HEA coating. It is known that the corrosion resistance of carbides is generally better than that of HEAs [47]. With increasing ZrC content, the carbide content increases in the coating. Therefore, the corrosion resistance of the CrMnFeCoNi HEA coating is gradually enhanced with increasing ZrC content.

**Figure 14:**
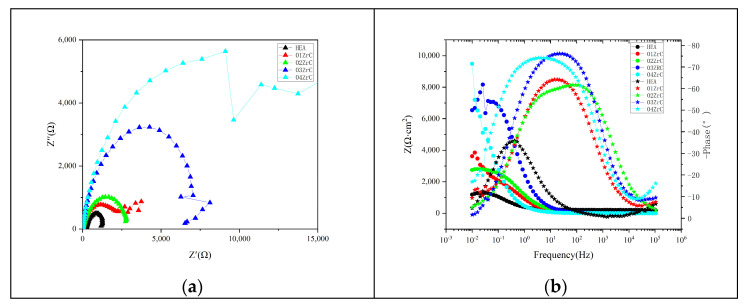
EIS of HEA and XZrC coatings: (**a**) Nyquist; (**b**) Bode.

Figure 14 shows the electrochemical impedance spectroscopy (EIS) of five HEA coatings tested in 1 mol/L NaCl solution. It can be seen from the Nyquist plot that the radius of the capacitive reactance arc gradually increases as the ZrC content increases (Figure 14a). This indicates that ZrC can effectively increase the resistance of the coating. Furthermore, the resistance of the coating increases with the addition of more ZrC. In other words, as the ZrC content increases, the electron transfer of the coating becomes more difficult, which leads to an improvement in the corrosion resistance of the coating.

**Figure 15:**
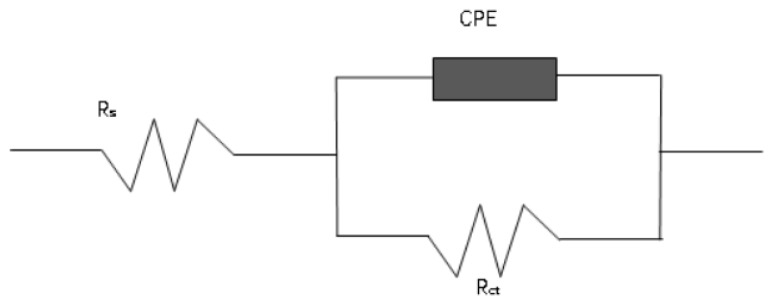
Equivalent circuit of HEA and XZrC coatings.

As can be seen from the Bode plot (Figure 14b), the phase angle curves of the five coatings all exhibit the characteristics of a single spike and peak valley, indicating the presence of only one time constant. Combined with the curve of the Nyquist diagram, the electrochemical corrosion model of the coating can be characterized by the equivalent circuit shown in Figure 15. Rs represents the solution resistance. Rct represents the charge transfer resistance in the electrode double layer, which is the equivalent polarization resistance. CPE represents the capacitance in the circuit. The equivalent circuit is fitted using the data analysis module of the potensiostat/galvanostat, and the results are presented in Table 9. It can be seen that the equivalent polarization resistance gradually increases with the increase in ZrC content. This indicates that the corrosion resistance of the coating gradually improves with an increase in ZrC content.

## 4. Conclusions

In this study, CrMnFeCoNi HEA coatings with different ZrC contents are prepared by a PTA process, and the effects of the ZrC content on the crystal structure, microstructure, composition, hardness, friction properties, and corrosion resistance of the CrMnFeCoNi HEA coatings are investigated. Moreover, the following conclusions are drawn.

1. The microstructure of HEA and coatings with varying ZrC contents consist of dendrites that are perpendicular to the substrate. The crystal structures of the CrMnFeCoNi HEA coatings with different ZrC contents are FCC. The results indicated that the addition of ZrC did not change the microstructure and morphology of the HEA coating. This is due to the fact that ZrC mainly exists in the CrMnFeCoNi HEA coating as a second phase.
2. The hardness of the CrMnFeCoNi HEA coating increases gradually with increasing ZrC content, and the hardness of the 01ZrC coating is approximately 20% higher than that of the HEA. However, the increase rate of the hardness decreases when the content of ZrC is more than 1 wt%.
3. ZrC had little effect on improving the wear resistance of the CrMnFeCoNi HEA coating. The addition of ZrC did not change the wear mechanism of the CrMnFeCoNi HEA coating. In every case, the wear was a combination of adhesive wear and abrasive wear. The wear resistance of the CrMnFeCoNi HEA coating showed a trend of first increasing, then decreasing, and finally increasing with increasing ZrC content. Although the increase in hardness improved the wear resistance of the 01ZrC coating, the improvement was not remarkable.
4. ZrC can effectively improve the corrosion resistance of the CrMnFeCoNi HEA coating, and the corrosion resistance of the CrMnFeCoNi HEA coating increases with increasing ZrC content. The annual corrosion rate of the 04ZrC coating in a 1 mol/L NaCl solution is only 21.5% of that of the HEA.

Consequently, the addition of ZrC to the FeCoCrNiMn high-entropy alloy coating holds promise for applications in corrosion resistance, particularly in oceanic environments.

## Figures and Tables

**Table 1:** Chemical composition of the Q235 substrate material (wt%).

C	Mn	Si	S	P	Fe
≤0.22%	≤1.4%	≤0.35%	≤0.050%	≤0.045%	Bal.

**Table 2:** Chemical composition of the CrMnFeCoNi high-entropy alloy (wt%).

Cr	Mn	Fe	Co	Ni
19.52%	20.86%	20.26%	19.49%	19.87%

**Table 3:** Process parameters of plasma cladding.

Carrier Gas Flow Rate (L/min)	Plasma Gas Flow Rate (L/min)	Powder Feed Rate (g/min)	Electric Current (A)	Deposition Speed (mm/min)	Working Distance to Substrate (mm)
5	5	50	180	300	10

**Table 4:** Parameters of the wear experiment.

Applied Load (g)	Time (min)	Experimental Temperature (°C)	Rotational Speed (r/min)	The Radius of the Sliding Wear Track (mm)
200	30	25	200	3

**Table 5:** Electrochemical experimental parameters.

EIS Test Method	Frequency Range (Hz)	Disturbance Voltage (mV)	Polarization Curve Test Method	Voltage Range (V)	Scanning Speed (mV/s)
Impedance-frequency scanning	10^−2^–10^−5^	10	Potentiodynamic polarization	−2~2	1

**Table 6:** Phase content in XRD analysis (%).

	FCC	ZrC	LAVES
HEA powder	100	0	0
HEA	100	0	0
01ZrC	95.3	4.7	0
02ZrC	94.4	5.6	0
03ZrC	93.8	6.2	0
04ZrC	88.8	6.3	4.9

**Table 7:** EDS analysis results of the CrMnFeCoNi and ZrC high-entropy alloy coating (wt.%).

Point	Cr	Mn	Fe	Co	Ni	Zr	C
A	4.16	6.52	3.01	2.56	2.70	62.60	18.46
B	16.89	22.89	18.23	17.93	21.99	0	1.89
C	0.56	0.56	0.58	0.43	0.41	64.54	32.27
D	17.11	15.27	22.02	18.81	17.68	3.66	5.45
E	1.63	0	2.93	0	0	64.40	29.38
F	18.68	17.04	21.79	19.84	17.82	0.01	4.83
G	4.24	5.19	8.04	3.35	3.18	51.07	22.93
H	18.22	15.64	20.48	17.54	15.68	5.48	6.98

**Table 8:** Electrochemical parameters of the high-entropy alloys with different ZrC contents.

Specimen	Rp (Ω/cm^2^)	E_corr_ (V)	I_corr_ (A/cm^2^)	Corrosion Rate (mm/a)
HEA	1076.6	−0.99367 ± 0.05	1.6673 × 10^−5^ ± 0.3 × 10^−5^	0.1956 ± 0.005
01ZrC	1962	−0.97638 ± 0.05	9.3374 × 10^−6^ ± 0.5 × 10^−6^	0.1095421 ± 0.005
02ZrC	2240	−0.96184 ± 0.05	8.0538 × 10^−6^ ± 0.5 × 10^−6^	0.094269 ± 0.005
03ZrC	7488.2	−0.58537 ± 0.05	2.4038 × 10^−6^ ± 0.5 × 10^−6^	0.024358 ± 0.005
04ZrC	18027	−0.44554 ± 0.05	9.9848 × 10^−7^ ± 0.5 × 10^−7^	0.011731 ± 0.005

**Table 9:** EIS parameters of HEA and XZrC coatings.

	Rs (Ω·cm^2^)	CPE	Y_0_ (S·Ω^−1^·cm^−2^)	Rt (Ω·cm^2^)
HEA	5.916	0.74374	9.4613 × 10^−5^	1286
01ZrC	7.597	0.75964	2.3709 × 10^−5^	3022
02ZrC	4.511	0.73601	1.6096 × 10^−5^	3452
03ZrC	6.291	0.86851	7.1758 × 10^−5^	7524
04ZrC	13.66	0.84460	5.6212 × 10^−5^	13,527

## Data Availability

Data are contained within the article.
